# Editorial to “Performance of the novel ANTWERP score in predicting heart function improvement after atrial fibrillation ablation in Asian patients with heart failure”

**DOI:** 10.1002/joa3.13178

**Published:** 2024-10-24

**Authors:** Yasushi Mukai

**Affiliations:** ^1^ Division of Cardiology Fukuoka Red Cross Hospital Fukuoka Japan

**Keywords:** atrial fibrillation, heart failure, left ventricular function

A number of studies have shown that sinus rhythm restoration by catheter ablation for atrial fibrillation (AF) elicits a recovery of left ventricular (LV) systolic function in patients with AF and heart failure.^1^ However, the extent of LV function improvement may vary among patients depending on their clinical background and disease progression. It is favorable if the recovery of LV function is predictable in prior to undergoing AF ablations. A recent European cohort study demonstrated that a simple scoring of clinical factors, called ANTWERP score, is highly useful in predicting LV function recovery in patients with heart failure undergoing AF ablation.^2^ The ANTWERP score consists of four simple clinical variables including QRS width, existence of known etiology of heart failure, type of AF, and severe left atrial dilatation. In a new report in the present issue of the journal, Ling et al. conducted a validation study of the ANTWERP score in an Asian population of AF patients with heart failure.^1^ The study showed that the ANTWERP score was similarly reliable in predicting the LVEF recovery in an Asian group of patients.

It is easily understandable that myocardial damage caused by organic heart diseases such as cardiomyopathies or ischemic heart disease cannot be healed with cardiac rhythm management alone. On the other hand, AF‐induced myocardial damage can be irreversible if the disease process is long‐lasting so as to elicit irreversible LV myocardial damage and fibrosis. In a study of cardiac MRI, the presence of an extensive gadolinium enhancement, a marker of LV fibrosis, was associated with a poor recovery of LV function in patients with AF and heart failure undergoing ablations.^3^ Looking back the ANTWERP score, a wider QRS width and left atrial dilatation may be simply reflecting the irreversible LV myocardial damage and duration of AF persistence, respectively. It is useful that LV functional recovery after AF ablation is predictable by such a simple scoring of the clinically available information. However, the recovery of LV systolic function per se may not totally determine the total clinical benefit of AF ablation and sinus rhythm restoration. Thus, clinical decision‐making whether or not undergoing AF ablation should be under a comprehensive consideration.

Some racial differences can be observed in morbidity and mortality in patients with AF. As discussed in the present study, Black patients with AF tend to have higher mortality and stroke rates than other races, which may partly be related to economic or insurance status. However, it was noted that for ethnic minorities enrolled in the North American prospective cohort, the clinical benefits of catheter ablation seemed to be more significant in comparison with nonminority cohort.^4^ There is only little information regarding the relative clinical benefits of AF ablation in Asian population in comparison with other ethnicities, whereas a number of excellent study results of AF ablation reported from the Asia‐Pacific region may warrant great clinical benefits of AF ablation in Asian patients with heart failure. In terms of recovery of LV systolic function after ablation, predicting factors may be different among races and regions because patient background can be different, that is, the rate of ischemic heart disease. However, in the present study, the ANTWERP score was similarly reliable in predicting LVEF recovery in the studied Asian patient group.

In the present study, the rate of recurrent atrial arrhythmia was comparable between the responder group and the nonresponder group. Together with the recent clinical trials, a reducing AF burden is associated with favorable clinical outcomes, including LV functional recovery, lower mortality, and HF hospitalization rates, even if some AF recurrence exists. Therefore, reducing AF burden is an important and acceptable therapeutic goal in managing AF, especially in heart failure patients. Such consideration is important to assess the results of clinical trials and studies, and thus clinical impact of AF ablation. Obviously, the clinical benefit of AF ablation should be considered not only with atrial tachyarrhythmia‐free achievement, considering the progressive and relapsing feature of AF.

## CONFLICT OF INTEREST STATEMENT

The author declares no conflict of interest for this article.
